# Editorial: Insights in research methods and advances in epidemiology: 2022

**DOI:** 10.3389/fepid.2023.1274569

**Published:** 2023-09-04

**Authors:** Ralph Brinks

**Affiliations:** Department for Medical Biometry and Epidemiology, Faculty of Health, University Witten, Herdecke, Germany

**Keywords:** epidemiological methods, epidemiological modeling, data analysis—epidemiology, meta research, regression analyses

**Editorial on the Research Topic** Insights In Research Methods and Advances in Epidemiology: 2022

More than a decade ago, Chubb and Jacobsen stated “Many epidemiologists may think that statistical regression is the only modeling technique available for the epidemiologist's toolkit” (1) and, indeed, the manifold applications of regression play a predominant role in epidemiology. Although Chubb and Jacobsen could not foresee the fast evolution of the field of causal inference in epidemiology during the last few years, the predominant role of a few statistical techniques in such a diverse field like epidemiology is a clear indication that, compared with other scientific domains, epidemiology is widely uncharted territory. Of course, the discipline of epidemiology is young compared with other domains like medicine and physics, in which great achievements have already been made several centuries ago. However, epidemiological knowledge and appropriate methodological skills and techniques are vitally important in a globalized world. The SARS-CoV-2 pandemic in the past years made this painfully explicit. For example, the question of whether the positive effects of lockdowns during the pandemic outweighed the negative effects on health-related outcomes is still highly disputed. Moreover, traditionally grown boundaries between infectious disease epidemiology and chronic disease epidemiology were crossed by the fact that chronically ill people have been at a higher risk of death from COVID.

The demand for a variety of methodological skills and their improvements was among the reasons why former Specialty Chief Editor of the Methods and Advances in Epidemiology section of Frontiers in Epidemiology, Rolf Groenwold, has chosen the Research Topic *Insights In Research Methods and Advances in Epidemiology: 2022*.

The first published contribution to the Research Topic, *Analysis of Recurrent Times-to-Clinical Malaria Episodes and Plasmodium falciparum Parasitemia: A Joint Modeling Approach Applied to a Cohort Data* (Stanley et al.), deals with recurrent episodes of malaria, which are analyzed by “joint models”. Joint models are a timely topic in medical statistics and typically consist of two components: a continuous repeatedly measured longitudinal outcome *Y*, and a time-to-event component described by a hazard rate *λ*. The two models are linked by a set of subjects' covariates *X*, usually called a trajectory. All variables, *Y*, *λ*, and *X*, generally depend on time *t*. The article defines *Y* as the concentration of parasites in the blood and uses the rate *λ* to describe the rate of clinical symptoms of recurrent infections. Covariates are age, gender, season, and the use of bed nets. The original data in the article stem from a malaria study conducted in Malawi from 2014 to 2015. Apart from the original data analysis, the article gains extensive and fruitful insights through simulations about variations in sample size, the length of follow-up time, the percentage of censored data, and the correlation between the two components of the joint model.

The second published article of the Research Topic, *Modeling non-linear relationships in epidemiological data: The application and interpretation of spline models* (Schuster et al.), deals with modeling non-linear associations between exposure and outcomes by utilizing spline functions. Splines are low-order polynomials defined on subintervals meeting smoothness conditions at the start and endpoints of the subintervals. Using an example data set about measures for body fat and respiratory fitness from the Amsterdam Growth and Health Longitudinal Study, the performance of different curve fitting methods is compared in terms of the explained variance (similar to the coefficient of determination) and interpretability of the estimates. In the example data, the cubic spline outperforms the other methods with respect to the explained variance but is slightly less simple to interpret. They provide practical hints for using the spline models in common statistical software programs.

The aim of the third published article in the Research Topic, *Development of a gender score in a representative German population sample and its association with diverse social positions* (Wandschneider et al.), is to appropriately deal with gender as a social construct in epidemiology. Data about more than 20,000 people in the German Socioeconomic Panel are used to explore gendered social practices and their variation across social groups. In their article, the authors develop a gender score to explore drivers of heterogeneity in feminine-masculine social practices. It is shown that those items reflecting family and household structures obtain the highest weights in the gender score.

The fourth published article, *It’s time! Ten reasons to start replicating simulation studies* (Lohmann et al.), addresses the important aspect of replicability. Many scientific domains suffer from the reproducibility crisis, in which the results of many scientific studies are difficult or even impossible to reproduce. As a consequence, sometimes the quality and integrity of research is questioned. As reproducibility is a basic principle of the scientific method, the author team argues that simulation studies should not be exempt from attempts to replicate findings. As simulations analysis and their interpretation oftentimes are not neutral or self-evidently correct, the research community must be able to check and re-run the simulations. A very striking argument is that compared with empirical data, simulations are relatively easy to replicate if the source code of the tools for data generation and analysis is provided.

In the final article of the Research Topic, *Measuring educational neglect using the Q method: A model based on the burden of disseminated tungiasis* (Martins et al.), medical education about Tungiasis is explored. Tungiasis is a rare tropical disease caused by the sand flea *Tunga penetrans*. Students from the life sciences (mostly medicine) and healthcare professionals from Brazil were surveyed. The authors apply the Q methodology to explore the viewpoints of the students and professionals about the prevention and treatment of Tungiasis. The Q methodology is similar to the technique of factor analysis, which is better known in the field of statistics than the Q method. While the other four articles from the Research Topic primarily deal with quantitative data and simulations, the last article in the series deals with important qualitative data.

The five articles in the Research Topic follow a nice line, starting with a quantitative analysis of data about Malaria and ending with a qualitative analysis of data from another infectious disease, Tungiasis. In between, we have contributions about chronic conditions, gender-specific practices, and replicability, which reflect the variety of methodological approaches in epidemiology.
